# Assessment of pain location according to different types of bruxism

**DOI:** 10.22514/jofph.2025.016

**Published:** 2025-03-12

**Authors:** Burcu Anasız Kıranatlı, Özer İşisağ

**Affiliations:** ^1^Department of Prosthodontics, Faculty of Dentistry, Afyonkarahisar Health Sciences University, 03030 Afyonkarahisar, Turkey

**Keywords:** Pain, Temporomandibular disorder, Bruxism, Teeth clenching, Teeth grinding

## Abstract

**Background**: This study aimed to assess the influence of bruxism types and frequencies on pain localisation in individuals with temporomandibular disorders (TMD). **Methods**: The study participants included 100 TMD patients. Participants consented to undergo clinical evaluations based on the Diagnostic Criteria for Temporomandibular Disorders Assessment Instruments Protocol (DC/TMD). Pain was determined by palpating the temporal and masseter muscles and the temporomandibular junction (TMJ). The oral behavior checklist (OBC) was used in DC/TMD to assess participants’ risk for parafunctional movements and types of bruxism. The parafunctional risk assessment was performed with the assessment method reported by The International Research Diagnostic Criteria for Temporomandibular Disorders (RDC/TMD) Consortium Network. **Results**: Most participants are at high risk for parafunction. There was a statistically significant relationship between masseter, temporal and TMJ pain and parafunctional movements (*p* < 0.05). Masseter pain on palpation showed significant relationships with sleep bruxism and awake grinding frequency (*p* < 0.05). Temporal muscle pain on palpation showed significant relationships with the presence and frequency of awake clenching (*p* < 0.05). A significant correlation exists between TMJ pain on palpation and awake grinding and clenching and with awake clenching frequence (*p* < 0.05). **Conclusions**: Based on their frequency and presence, different types of bruxism may be associated with different pain symptoms.

## 1. Introduction

TMDs are musculoskeletal disorders affecting the TMJ, masticatory system, and related tissues [1, 2, 3, 4]. TMD is a multifactorial disorder, with no single culprit [5, 6, 7]. Five factors should be considered when evaluating the aetiology of TMD: occlusal conditions, trauma, emotional stress, deep pain source, and parafunctional activity [8, 9, 10].

TMD symptoms include pain and tenderness in the head and neck muscles, noise from the TMJ region, and restriction or deviation in mandibular movements. Additionally, headaches, ear pain, tinnitus, tooth wear, and mobility may be present [11, 12, 13]. All age groups can experience TMD symptoms. TMD symptoms are more common in females than males [14, 15, 16, 17, 18, 19], while prevalence is low in younger age groups.

Having experienced pain at an early age, it is subjective and derived from personal experiences. Pain perception is usually influenced by genetics, gender, and mental processes such as feelings and beliefs. Consequently, pain affects patients’ quality of life in a significant way [20]. TMD can lead to painful and functional impairment of the stomatognathic system, as the temporomandibular joints and related muscles are essential for various daily activities, including eating, speaking and facial expressions [5, 21, 22]. Parafunctional movements may contribute to or exacerbate related disorders such as pain and limited mouth opening [23, 24].

Bruxism is a repetitive jaw-muscle activity characterized by teeth clenching or grinding during wakefulness or sleep [25, 26, 27]. In the last edition of the Glossary of Prosthodontic Terms, bruxism is defined as “A repetitive jaw-muscle activity characterized by clenching or grinding of the teeth and/or bracing or thrusting of the mandible; it has two distinct circadian manifestations relating to whether it occurs during sleep (sleep bruxism) or during wakefulness” [28]. There is some evidence that it is associated with TMD and causes a variety of pain symptoms and functional limitations. Inconsistencies may be due to the lack of clear definitions of different bruxism patterns (*e.g.*, teeth clenching or grinding, nocturnal bruxism, daytime bruxism, *etc.*) in the studies conducted [29, 30, 31, 32]. A meta-analysis of the global prevalence of bruxism found that the overall prevalence (both during sleep and while awake) is 22.22%. Sleep bruxism is prevalent at 21%, and awake bruxism is prevalent at 23%. According to polysomnography, 43% of people suffer from sleep bruxism [33]. It is essential for the physician to determine the etiologic factors and differentiate the factors that contribute to the current clinical condition to treat TMD effectively [8, 34]. This study aimed to assess the influence of bruxism types on pain localization in TMD patients. Bruxism types were hypothesized not to affect pain localization in the study’s null hypothesis.

## 2. Material and methods

Study participants were selected from individuals who presented to Afyonkarahisar Health Sciences University, Faculty of Dentistry, Department of Prosthodontics between 16 May 2022 and 16 June 2023 with a chief concern about jaw pain or were diagnosed with TMD. The ethical approval (Decision dated 13 May 2022 and number 2022/298) of this study was approved by the Non-Interventional Ethics Committee of Afyonkarahisar Health Sciences University. This study was conducted after obtaining written informed consent from patients according to the Declaration of Helsinki.

This study included participants aged 15–85 with symptoms of TMD (Spontaneous or palpable pain in the temporomandibular region, TMJ noise, limited mouth opening, and mandibular deviation or deflection when opening the mouth) who could read and write in Turkish. Individuals with TMD symptoms were not subdivided into TMD subgroups. Participants without consent, unable to communicate and without TMD symptoms were excluded. Keeping the age range high ensured a large sample size.

The G*Power package (G*Power Ver. 3.0.10, Franz Faul, University of Kiel, Kiel, SH, Germany) was used to calculate the sample size. The *t*-test family was used to compare two independent groups, with a power set at 0.90 and effect size set at 0.59, according to Cohen’s *d*. For comparisons between more than 2 groups, one-way analysis of variance test from the F test family was used, with a power of 0.80, and an effect size of 0.115 according to the partial eta squared (η^2^) value. Fisher’s exact test from the exact test family was used to evaluate the 2 × 2 crosstab analysis, with a power of 0.80, and the effect size of 3.55 according to the odds ratio (OR) value. Based on all analyses, 100 participants were selected.

One researcher evaluated and examined participants, while two analysed the data. Clinical evaluations were conducted on consenting individuals according to the DC/TMD protocol. Palpation examination was used to assess participants’ pain findings. According to the DC/TMD 2013 protocol, 1 kg of pressure was applied for 5 seconds to the temporal (three sites: anterior, middle, and posterior) and masseter muscles (three sites: superior, middle and inferior) as well as the TMJ (at the lateral pole and around the lateral pole). A force meter was used to calibrate the fingers before each palpation examination. Whenever a patient reported pain beyond the palpable muscle or joint, it was classified as referred pain. Nevertheless, if the pain was limited to the muscle or joint boundaries, it was not considered referred. If the patient experienced pain, the examiner asked if it was similar to pain experienced before the visit. If so, it was classified as familiar pain.

OBC in DC/TMD was used to assess participants’ risk based on their parafunctional movements and to determine the types of bruxism they suffered. Parafunctional risk was assessed using the OBC scoring method reported by the International RDC/TMD Consortium Network. Scoring was calculated as the sum of item numbers with a non-zero response or a weighted sum (*i.e.*, summing endorsement frequencies of the relevant items). Comparing people with chronic TMD to those without, an OBC summary score of 0–16 represents normal behavior. Scores of 17–24 occur twice as often in TMD patients, and 25–62 occur 17 times more often. Only a score in the 25–62 range is considered a risk factor for TMD. Individuals were asked to indicate bruxism during sleep as never, <1 night/month, 1–3 nights/month, 1–3 nights/week, 4–7 nights/week, as well as clenching or grinding during wakefulness as none, a little, some, most and all of the time. Data analysis was performed using Statistical Package for the Social Sciences (SPSS) (Version 22, IBM Corp., Armonk, NY, USA). To compare paired means, independent sample *t*-tests and Mann-Whitney U tests were used. Cohen’s *d*-effect size was calculated for statistically significant comparisons. A one-way Analysis of variance (ANOVA) test was used for comparisons involving more than two groups, and Eta-squared (η^2^) was used to measure effect size. For η^2^, the thresholds are: small 0.01, medium 0.06, large 0.14 [35]. Cross-table was used to determine categorical variables. A Pearson Chi-Square test was applied when the sample size assumption (expected value >5) was met, and a Fisher’s Exact test was applied when it was not. The effect size was evaluated with OR. For OR, the thresholds are: small 1.44, medium 2.48, large 4.27 [36]. *p* < 0.05 indicates statistically significant differences.

## 3. Results

Table 1 shows the risk status of individuals for parafunction and bruxism types distribution according to the OBC inventory. The majority of participants were at high risk of parafunction. Table 2 shows the relationship between OBC scores and palpation pain in masseter, temporal muscles, and TMJ. Individuals with palpation pain in temporal and masseter muscles and TMJ had higher OBC scores. There were statistically significant relationships between OBC scores and temporal pain at a medium effect size (*p* = 0.002, Cohen’s *d* = 0.702), masseter pain at a high effect size (*p* = 0.001, Cohen’s *d *= 0.869) and TMJ pain at a medium effect size (*p* = 0.001, Cohen’s *d* = 0.703).

**Table 1.** The risk status of individuals according to the OBC Inventory and the distribution of bruxism types.

OBC inventory		Percentage %
Risk status		
	No risk	1
	Low risk	34
	High risk	65
Bruxism types		Frequency
Sleep bruxism		
	None of the time	10
	<1 Night/Month	17
	1–3 Nights/Month	17
	1–3 Nights/Week	17
	4–7 Nights/Week	39
Awake grinding		
	None of the time	44
	A little of the time	19
	Some of the time	27
	Most of the time	8
	All of the time	2
Awake clenching		
	None of the time	26
	A little of the time	13
	Some of the time	31
	Most of the time	20
	All of the time	10

*OBC: oral behaviour checklist.*

**Table 2.** The relationship between parafunctional movements and pain in the masseter and temporal muscles and TMJ.

n			Mean	SD	Median	Test Statistic	*p*	Cohen’s *d*
Temporal Pain								
	Yes	43	33.07	13.26	34.00	−3.114	0.002*	0.702
	No	57	25.54	10.89	27.00			
Masseter Pain								
	Yes	81	30.67	12.39	31.00	402.000	0.001*	0.869
	No	19	20.74	9.48	20.00			
TMJ Pain								
	Yes	66	31.62	12.47	33.50	−3.330	0.001*	0.703
	No	34	23.26	10.64	22.50			

*p < 0.05. TMJ: temporomandibular junction; SD: standard deviation.

### 3.1 Sleep bruxism

Sleep bruxism frequency and palpation pain distribution among participants were analysed by cross-table (Table 3). Pearson’s chi-square and Fisher’s exact tests showed no statistically significant relationships between sleep bruxism frequency and temporal pain (*p* = 0.434), masseter pain (*p* = 0.129) and TMJ pain (*p* = 0.240). The distribution and cross-table between sleep bruxism and palpation pain are shown in Table 4. In contrast to sleep bruxism frequency, a statistically significant relationship was found between masseter pain and bruxism at a high effect size (*p* = 0.020, odds ratio (OR) = 5.429).

**Table 3.** Cross-table analyses for the frequency of sleep bruxism and the presence of pain.

Pain Presence		None of the time, n (%)	<1 Night/Month, n (%)	1–3 Nights/Month, n (%)	1–3 Nights/Week, n (%)	4–7 Nights/Week, n (%)	Test Statistic	*p*
Temporal Pain								
	Yes	2 (20.0)	9 (52.9)	7 (41.2)	6 (35.3)	19 (48.7)	3.799	0.434
	No	8 (80.0)	8 (47.1)	10 (58.8)	11 (64.7)	20 (51.3)		
Masseter Pain								
	Yes	5 (50.0)	13 (76.5)	14 (82.4)	15 (88.2)	34 (87.2)	6.933	0.129
	No	5 (50.0)	4 (23.5)	3 (17.6)	2 (11.8)	5 (12.8)		
TMJ Pain								
	Yes	4 (40.0)	11 (64.7)	11 (64.7)	10 (58.8)	30 (76.9)	5.502	0.240
	No	6 (60.0)	6 (35.3)	6 (35.3)	7 (41.2)	9 (23.1)		

*TMJ: temporomandibular junction.*

**Table 4.** Analysing the presence of bruxism and pain regions with cross-table analysis.

Bruxism presence		Temporal Pain		Masseter Pain		TMJ Pain	
		Yes	No	Yes	No	Yes	No
Sleep bruxism							
	Yes	41	49	76	14	62	28
	No	2	8	5	5	4	6
		*p* = 0.181		*p* = 0.020*, OR = 5.429		*p* = 0.085	
Awake grinding							
	Yes	27	29	49	7	42	14
	No	16	28	32	12	24	20
		*p* = 0.235		*p* = 0.062		*p* = 0.032*, OR = 2.500	
Awake clenching							
	Yes	37	37	60	14	56	18
	No	6	20	21	5	10	16
		*p* = 0.017*, OR = 3.333		*p* = 1.000		*p* = 0.001*, OR = 4.978	

*p < 0.05. TMJ: temporomandibular junction; OR: odds ratio.

### 3.2 Awake grinding

Awake grinding frequency and palpation pain distribution were analysed by cross-table (Table 5). Fisher’s exact tests found a statistically significant relationship between teeth grinding frequency and masseter pain (*p* = 0.023). According to one-way ANOVA test, which was performed to evaluate the effect size in addition to statistical significance, a statistically significant relationship was found between teeth grinding frequency and masseter pain at medium effect size (*p* = 0.041, η^2^ = 0.098) (Table 6). The distribution and cross-table analysis between grinding and palpation pain are shown in Table 4. In contrast to grinding frequency, TMJ pain (*p* = 0.032, OR = 2.500) showed a statistically significant relationship with grinding at medium effect size.

**Table 5.** Cross-table analyses for the frequency of awake grinding and the presence of pain.

Pain Presence		None of the time, n (%)	A little of the time, n (%)	Some of the time, n (%)	Most of the time, n (%)	All of the time, n (%)	Test Statistic	*p*
Temporal Pain								
	Yes	16 (36.4)	6 (31.6)	14 (51.9)	6 (75.0)	1 (50.0)	6.095	0.162
	No	28 (63.6)	13 (68.4)	13 (48.1)	2 (25.0)	1 (50.0)		
Masseter Pain								
	Yes	32 (72.7)	14 (73.7)	26 (96.3)	8 (100.0)	1 (50.0)	10.362	0.023*
	No	12 (27.3)	5 (26.3)	1 (3.7)	0 (0.0)	1 (50.0)		
TMJ Pain								
	Yes	24 (54.5)	12 (63.2)	22 (81.5)	7 (87.5)	1 (50.0)	7.495	0.089
	No	20 (45.5)	7 (36.8)	5 (18.5)	1 (12.5)	1 (50.0)		

*p < 0.05. TMJ: temporomandibular junction.

**Table 6.** Evaluation of the relationships between bruxism frequencies and pain types according to one-way ANOVA test.

Pain regions and type of bruxism		*p*, η^2^
Temporal Pain		
	Sleep bruxism	0.446
	Awake grinding	0.200
	Awake clenching	0.020*, η^2^ = 0.114
Masseter Pain		
	Sleep bruxism	0.090
	Awake grinding	0.041*, η^2^ = 0.098
	Awake clenching	0.351
TMJ Pain		
	Sleep bruxism	0.246
	Awake grinding	0.117
	Awake clenching	0.002*, η^2^ = 0.165

*p < 0.05. TMJ: temporomandibular junction.

### 3.3 Awake clenching

Participants’ palpation pain distribution and clenching frequency were analysed using a cross-table (Table 7). According to Pearson’s chi-square and Fisher’s exact tests, there was a statistically significant relationship between clenching frequency and temporal muscle pain (*p* = 0.022) and temporomandibular joint pain (*p* = 0.002). One-way ANOVA test found that clenching frequency was significantly related to temporal pain with a medium effect size (*p* = 0.020, η^2^ = 0.114) and TMJ pain with a large effect size (*p* = 0.002, η^2^ = 0.165) (Table 6). Table 4 shows the distribution and cross-table analysis of clenching and palpation pain. It was also found that clenching was significantly correlated with temporal muscle pain at medium effect size (*p* = 0.017, OR = 3.333). Conversely, there was a significant relationship between temporomandibular joint pain at large effect size (*p* = 0.001, OR = 4.978).

**Table 7.** Cross-table analyses for the frequency of awake clenching and the presence of pain.

Pain Presence		None of the time, n (%)	A little of the time, n (%)	Some of the time, n (%)	Most of the time, n (%)	All of the time, n (%)	Test Statistic	*p*
Temporal Pain								
	Yes	6 (23.1)	5 (38.5)	12 (38.7)	13 (65.0)	7 (70.0)	11.476	0.022*
	No	20 (76.9)	8 (61.5)	19 (61.3)	7 (35.0)	3 (30.0)		
Masseter Pain								
	Yes	21 (80.8)	9 (69.2)	25 (80.6)	19 (95.0)	7 (70.0)	4.930	0.274
	No	5 (19.2)	4 (30.8)	6 (19.4)	1 (5.0)	3 (30.0)		
TMJ Pain								
	Yes	10 (38.5)	7 (53.8)	23 (74.2)	18 (90.0)	8 (80.0)	16.577	0.002*
	No	16 (61.5)	6 (46.2)	8 (25.8)	2 (10.0)	2 (20.0)		

*p < 0.05. TMJ: temporomandibular junction.

## 4. Discussion

This study aimed to investigate and evaluate the influence of bruxism types on pain localization in TMD patients. It was determined that the pain location varied depending upon the specific type of bruxism observed, thus rejecting the null hypothesis.

A major challenge in clinical trials related to TMD is defining aetiological factors, which makes standardizing participants difficult. In this context, a simple, understandable and reliable inventory is crucial to ensuring the reliability of the studies and evaluating and comparing the results. DC/TMD, developed by the International Network for the Methodology of Orofacial Pain and Related Disorders (INFORM) is used for this purpose [37, 38, 39, 40, 41]. DC/TMD inventory was used in this study to assess participants. Clenching or grinding teeth while sleeping or awake, nail-biting, chewing gum, and other parafunctional habits can cause stress to the mandible [42, 43]. TMD can be caused by parafunctional habits and muscle tension [44, 45, 46]. Chow and Cioffi [47] reported that excessive masticatory muscle overload may cause pain symptoms if the masticatory muscles are used to clench or grind teeth, play with the tongue, cheeks, and lips, or hold or bite objects like hair, pipes, pens, or fingers between their teeth. Bruxist patients have also complained of muscle tension, stiffness, and pain in the face, ear, frontotemporal region [48]. Chen *et al*. [49] reported that patients with myofascial pain in the jaw, temples, face, preauricular area, or ear at rest or during function had 4 times more nonfunctional dental contact and stress levels when awake. This study examined a group of TMD patients. Most participants exhibited a high tendency to engage in parafunctional oral habits, and a significant relationship was found between OBC scores and palpation pain. Accordingly, parafunctional habits may be influential in the onset and progression of TMD.

Distinct associations were found between different bruxism patterns and palpation pain in specific anatomical regions. Individuals with a habit of awake grinding were found to experience pain in the masseter muscle and TMJ upon palpation. Conversely, individuals with awake clenching showed palpation pain in the temporal muscle and TMJ. The observed correlations are supported by several studies in the literature. Researchers found that teeth grinding accounts for approximately 60% of the muscle activity of the masseter muscle when examining the impact of various types of bruxism. Additionally, teeth clenching accounted for 37% of muscle activity [50]. An electromyographic (EMG) measurement was also performed in another study to assess muscle activity during swallowing and maximum clenching. EMG activity of the temporal muscle was better than that of the masseter muscle during these activities [51]. Bruxism patterns differed with respect to pain perception when palpated at different anatomical locations in the study. It may shed light on the mechanisms behind bruxism by increasing activity in specific muscle groups. Research and treatment approaches for bruxism could benefit from these implications. Studies have pointed out the challenge of detecting parafunctional movements in sleep, often because the individual is unaware of this disorder [52, 53]. The relationship between nocturnal bruxism and TMD has been a subject of ongoing debate. A study has proposed the use of standardized tools in nocturnal bruxism evaluation. It may be possible to resolve this debate by using these tools and to provide a more accurate assessment of the relationship between nocturnal bruxism and TMD [54]. The American Academy of Sleep Medicine (AASM) proposed widely accepted criteria for diagnosing sleep bruxism. The International Classification of Sleep Disorders (ICSD) 2014 update includes regular tooth-grinding sounds during sleep and one or more clinical signs and symptoms. Among them are abnormal tooth wear, morning jaw muscle pain, fatigue, temporal headaches, or jaw locking upon awakening, with reports of tooth grinding during sleep [55]. Bruxism assessment involves patient-reported symptoms, clinical examinations findings, and measurements such as EMG and polysomnography. By integrating these diverse sources of information, healthcare professionals can determine bruxism’s presence and nature [56]. In this study, although significant relationships were expected between sleep bruxism and all palpation pain (temporal, masseter and TMJ), only sleep bruxism and masseter pain were found to be significant. This may be because sleep bruxism was detected via subjective knowledge, different diagnostic techniques were not used, and no sleep bruxism was detected. Several limitations should be considered in this study. The sample size of the study was relatively small, which may impact its generalizability. The exclusion criteria also excluded factors such as medication use that exacerbates bruxism, trauma history, musculoskeletal disorders, and sleep apnoea. The diagnosis of bruxism was also based solely on subjective information provided by participants, without the use of additional diagnostic techniques such as polysomnography. Moreover, the results may be affected by the participants’ co-occurrence of awake and sleep bruxism. It is essential to acknowledge these limitations when interpreting study results.

## 5. Conclusions

Within the limitations of this study, it can be concluded that:

1. Parafunctional oral habits are significantly related to palpation pain.

2. There is a significant relationship between the presence of awake grinding and TMJ pain, and between frequency and masseter pain.

3. The presence and frequency of awake clenching showed significant relationships with temporal muscle pain and TMJ pain on palpation.

4. Although there was no significant relationship between the frequency of sleep bruxism and the pain on palpation examined in the study, the presence of sleep bruxism was significantly associated with masseter pain on palpation. However, the examination for sleep bruxism in this study was limited, which may lead to bias.

5. Different bruxism types may be associated with pain symptoms in different areas, depending on their frequency and presence.
